# Effective Protection Against Status Epilepticus Caused by Lithium–Pilocarpine: Combination of Midazolam and Lacosamide

**DOI:** 10.1002/brb3.70546

**Published:** 2025-05-11

**Authors:** Cumaali Demirtas, Metehan Akca, Ugur Aykin, Yunus Emre Surmeneli, Hava Yildirim, Mehmet Yildirim

**Affiliations:** ^1^ Department of Physiology, Hamidiye Faculty of Medicine University of Health Sciences İstanbul Türkiye; ^2^ Department of Physiology, Faculty of Medicine Tokat Gaziosmanpaşa University Tokat Türkiye; ^3^ Department of Medical Biology, Hamidiye Faculty of Medicine University of Health Sciences İstanbul Türkiye

**Keywords:** fosphenytoin, lithium–pilocarpine, midazolam, status epilepticus, valproic acid

## Abstract

**Aim:**

Status epilepticus causes the most severe condition related to epilepsy in terms of high mortality rate. Although status epilepticus treatment guidelines specify a treatment process based on three‐stage monotherapy, effective control cannot yet be achieved in all cases. In the presented study, with electrophysiological and behavioral tests, it was aimed to investigate the effectiveness of the combination of midazolam (MDZ), one of the most commonly used benzodiazepines in the first‐line treatment of status epilepticus, with the second‐line antiepileptics levetiracetam (LEV), lacosamide (LCM), valproic acid (VPA), and fosphenytoin (fPHT).

**Methods:**

A status epilepticus model was created with lithium–pilocarpine (5 mEq/kg–320 mg/kg) in adult male Sprague–Dawley rats with implanted electroencephalography (EEG) electrodes. MDZ (9 mg/kg) alone or in dual combinations with antiepileptic drugs (200 mg/kg LEV, 50 mg/kg LCM, 300 mg/kg VPA, 100 mg/kg fPHT) was injected i.p. to the experiment groups with status epilepticus. After video‐EEG recordings were taken from the rats during and after status, the effects of drug interactions on cognitive and motor behaviors were examined by applying behavioral tests (open field, Rotarod, radial arm maze, and passive avoidance).

**Results:**

Compared with the untreated status epilepticus group, it was determined that MDZ alone and the combination of four antiepileptic drugs administered with MDZ significantly reduced the mortality rate, spike frequency, and spike amplitude of epileptic seizures and suppressed epileptic seizures at certain levels (*p* < 0.01). Compared to MDZ monotherapy, it was determined that the mortality rate and spike frequency and amplitude decreased significantly in the MDZ + LCM group (*p* < 0.01), whereas on the other hand, mortality and spike frequency increased in the MDZ + LEV group (*p* < 0.01). No negative effects were observed in learning and memory in all treatment groups, but it was determined that the motor functions of the animals treated with MDZ + fPHT were impaired compared to both the control group without any treatment and the MDZ group (*p* < 0.01).

**Conclusion:**

In the status epilepticus model induced by lithium–pilocarpine, the combination of MDZ + LCM was found to be the most effective polytherapy option in reducing seizures and mortality. Additionally, it was observed that LEV, LCM, and VPA administered together with MDZ did not negatively affect both cognitive and motor functions.

## Graphical abstract

Graphical abstract of the study examining the effects of levetiracetam, lacosamide, valproic acid and fosphenytoin administered with midazolam on the spike frequency and amplitude of epileptiform activity in a lithium pilocarpine‐induced refractory status epilepticus model.

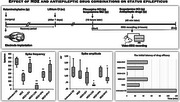

## 1 Introduction

Epilepsy is a neurological disease affecting up to 1% of the population (Hauser and Hersdorffer 1990). Although there are more than 20 antiepileptic drugs today, approximately 30% of epileptic seizures cannot be controlled with existing drugs (Regesta and Tanganelli 1999; French 2007). One of the most severe conditions related to epileptic seizures is status epilepticus. According to the latest evaluations, convulsive status epilepticus is defined as the persistence of clinical and/or electrographic seizure activity for 5 min or more or the absence of consciousness between two seizures (Brophy et al. 2012). Status epilepticus, which has an incidence of approximately 10–30/100,000, constitutes at least 1% of admissions to intensive care units (Sánchez and Rincon 2016). Status epilepticus has a mortality rate of 8%–65%, depending on age, medical comorbidity, the presence of nonconvulsive status epilepticus, and the cause of the seizures (Brophy et al. 2012). It has been reported that the 30‐day mortality of patients with generalized convulsive status epilepticus is in the range of 19%–27% (Towne et al. 1994; Treiman et al. 1998; Legriel et al. 2008). Prolonged seizures have been associated with higher mortality and worse clinical outcomes (Scholtes et al. 1994; Legriel et al. 2008).

Concerning the treatment of status epilepticus, current American and European guidelines recommend a stepwise approach based on the use of several different antiepileptic drugs (Meierkord et al. 2010; Brophy et al. 2012). The first recommended treatment option is a benzodiazepine administration (Silbergleit et al. 2012). At this stage, it has been stated that if intravenous (i.v.) access is provided, i.v. lorazepam (LZP) should be preferred. Otherwise, intramuscular (i.m.) midazolam (MDZ) is equally effective (Silbergleit et al. 2012). It has been shown that seizures will be easier to control when status epilepticus treatment is started earlier (Lowenstein and Alldredge 1993). To provide a more effective and rapid treatment in the early period, there are recommendations for using more than one drug in first‐line treatment (Alvarez and Rossetti 2016). Regarding the early treatment of status epilepticus, the potential use of levetiracetam (LEV), valproic acid (VPA), or lacosamide (LCM) as polytherapy together with a suitable benzodiazepine in patients who do not respond to benzodiazepine within 5 min has been recommended (Radhakrishnan 2016). Considering the guidelines in both the United States and Europe, LZP or MDZ as benzodiazepines are generally recommended for the first‐line treatment of status epilepticus, and phenytoin (PHT), phenobarbital (PB), VPA, or LEV are generally recommended for the second‐line treatment (Bhattacharjee and Hirsch 2015). However, there is not enough information in the literature regarding the potential for simultaneous and combined use of first‐line and second‐line antiepileptic drugs as polytherapy for the treatment of status epilepticus.

In the presented study, it was aimed to examine the interaction of MDZ, a benzodiazepine used in the first‐line treatment of status epilepticus, in combination with fosphenytoin (fPHT), VPA, LEV, or LCM in treatment of refractory status epilepticus model induced by lithium pilocarpine in Sprague–Dawley rats and to determine their potential for use in early polytherapy.

## 2 Materials and Methods

### 2.1 Animals

Fifty‐two male Sprague–Dawley rats, 10–12 weeks old with an average weight of 270 ± 30 g, were used in the experiments. The rats were bred and housed at the Hamidiye Experimental Animal Production and Research Laboratory of the University of Health Sciences (SBÜ), without food and water restrictions, at an ambient temperature of 21°C ± 3°C, 65%–70% humidity conditions, and under a 12‐h light–dark cycle.

### 2.2 Drugs and Chemicals

Lithium chloride (LiCl), pilocarpine, and scopolamine methylbromide (scopolamine‐Mbr) were purchased from Sigma (St. Louis, USA), and scopolamine hydrochloride (scopolamine‐HCl) and fPHT were purchased from ChemCruz (Dallas, USA). LCM was obtained from Sanovel A.Ş. (İstanbul, Türkiye). Commercially available drug preparations of MDZ (Sedever, Haver Ecza, İstanbul, Türkiye), VPA (Depakin, Sanofi Ltd.Ş., İstanbul, Türkiye), and LEV (Levetam, Polifarma İlaç A.Ş., Tekirdağ, Türkiye) were used. LCM was dissolved in 10% ethanol, and the remaining drugs and chemicals (LiCl, pilocarpine, scopolamine‐Mbr, scopolamine‐HCl, and fPHT) were dissolved in physiological saline (0.9% NaCl).

### 2.3 Experiment Design

**FIGURE 1:**
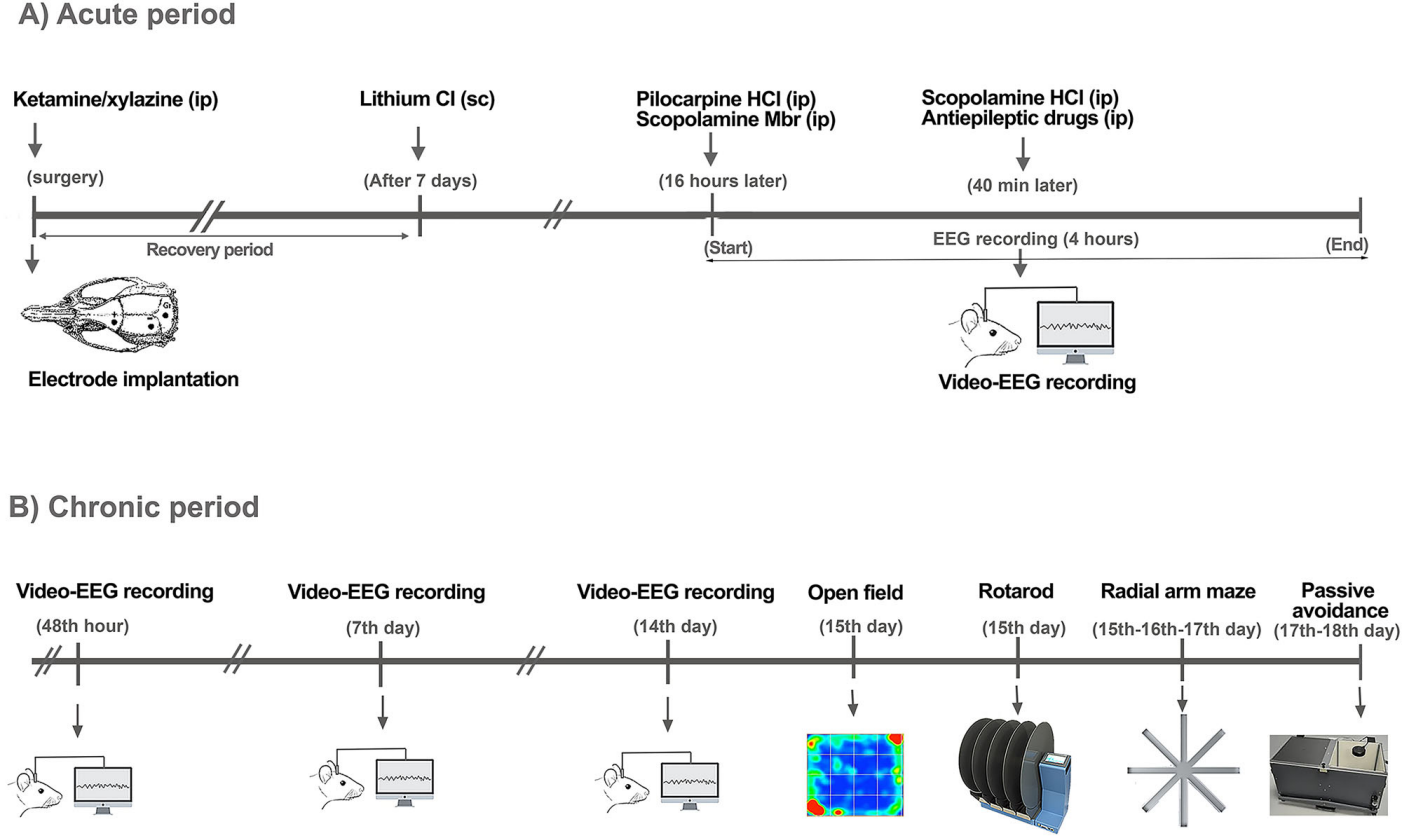
**Experimental design**. (A) After creating a status epilepticus model in the acute period, MDZ and antiepileptic drug combinations were applied, and video‐EEG recording was performed. (B) Behavioral tests were applied after video‐EEG recordings were made in the chronic period. MDZ: midazolam; seizures began approximately 7–8 min after pilocarpine injection. EEG, electroencephalography.

One week after electrodes were surgically implanted in the rats in groups other than the control group, a status epilepticus model was created, and antiepileptic drugs were administered. The experiments were carried out in two stages: the acute period, including creating the status epilepticus model and recording video‐electroencephalography (EEG), and the chronic period, including video‐EEG recording and motor‐cognitive performance tests for 2 weeks after status epilepticus. In the acute period, 5 mEq/kg LiCl was injected subcutaneously (s.c.), and 16 h later, 320 mg/kg pilocarpine and 1 mg/kg scopolamine‐Mbr were administered intraperitoneally (i.p.). Thirty minutes after the onset of status epilepticus in the animals, relevant drug and drug combinations, including MDZ, MDZ + LEV, MDZ + LCM, MDZ + VPA, and MDZ + fPHT, were injected i.p. route. At this stage, 10 mg/kg scopolamine‐HCl (i.p.) was also applied, which did not stop status epilepticus but cleared the excess pilocarpine from the environment (Niquet et al. 2017; Lumley et al. 2021). Video‐EEG monitoring was started 30 min before pilocarpine injection and was performed for a total of 4 h (Figure 1A).

The chronic phase included video‐EEG monitoring, seizure scoring, and behavioral tests conducted at 48 h, 7th, and 14th days following the establishment of the status epilepticus model with lithium–pilocarpine. The purpose of these assessments was to determine neuronal damage due to status epilepticus and drug side effects related to polytherapy. Additionally, on the 15th day following status epilepticus, open field and the Rotarod test were conducted, whereas the radial arm maze test was performed on Days 15–16–17, and the passive avoidance test on Days 17–18 (Figure 1B). When applying behavioral tests, the effort and stress to be exhibited in the tests were taken into consideration, and to minimize the effects of the tests on animal behavior, a test application order of open field, Rotarod, radial arm maze, and passive avoidance was planned. The first‐day procedures of the 3‐day radial arm maze test regarding habituation were completed 6 h after the Rotarod test was completed on the 15th day, the 2nd‐day tests regarding learning were completed on the 16th day, and the 3rd‐day procedures regarding the test phase were completed 6 h before the learning procedures of the passive avoidance test on the 17th day.

The experimental groups were designed as follows:

– control (*n* = 8): Physiological saline (i.p.), only behavior experiments;
– status (*n* = 8): Induction of status epilepticus with 5 mEq/kg LiCl (s.c.) + 320 mg/kg pilocarpine (i.p.);
– MDZ (*n* = 8): Status epilepticus + 9 mg/kg MDZ (i.p.);
– MDZ + LEV (*n* = 6): Status epilepticus + 9 mg/kg MDZ (i.p.) + 200 mg/kg LEV (i.p.);
– MDZ + LCM (*n* = 8): Status epilepticus + 9 mg/kg MDZ (i.p.) + 50 mg/kg LCM (i.p.);
– MDZ + VPA (*n* = 6): Status epilepticus + 9 mg/kg MDZ (i.p.) + 300 mg/kg VPA (i.p.);
– MDZ + fPHT (*n* = 8): Status epilepticus + 9 mg/kg MDZ (i.p.) + 100 mg/kg fPHT (i.p.).

### 2.4 Placement of EEG Electrodes

Animals were anesthetized with the combination of ketamine hydrochloride (90 mg/kg, i.p.)/xylazine hydrochloride (10 mg/kg, i.p.). The scalp was incised approximately 3 cm from the midline in the rostro‐caudal axis, and the soft tissue on the skull was removed. Two stainless‐steel screws were inserted into the cranium on each hemisphere to secure the EEG electrode and its holding acrylic to the skull. To place the tripolar EEG electrode (MS333/2A, P1 Technologies, Roanoke, VA), holes were drilled on the cranium at the determined coordinates with the help of a micromotor. One of the active electrode leads of the tripolar EEG electrode was placed on the left frontal cortex at AP +2.0 and L 3.5 mm relative to Bregma; the second active electrode was placed on the left parietal cortex at AP −6.0 and L 4.0 mm relative to Bregma, and also the reference electrode was placed on the cerebellum at AP −2 mm to Lambda (Paxinos and Watson 1998). After the tripolar EEG electrode tips were placed epidurally, they were fixed to the cranium with dental acrylic and stainless‐steel screws (Velioglu et al. 2017, 2018; Efendioglu et al. 2020). The animals were allowed to recover for 7 days before initiating the status epilepticus model and EEG recording (Figure 1A).

### 2.5 The Lithium–Pilocarpine Model of Status Epilepticus

To create the status epilepticus model, animals were injected with 5 mEq/kg LiCl (s.c.) 16 h before pilocarpine injection. LiCl was used to increase the effectiveness of pilocarpine (Jope and Morrisett 1986). To induce seizures related to status epilepticus, 320 mg/kg pilocarpine HCl (i.p.) and 1 mg/kg scopolamine‐Mbr (i.p.) were administered simultaneously. Scopolamine‐Mbr, a muscarinic antagonist that cannot cross the blood–brain barrier, was used to reduce peripheral cholinergic side effects such as bronchial secretion. Thirty minutes after the epileptic discharges related to status epilepticus started, 10 mg/kg scopolamine‐HCl (i.p.) was injected, which was a seizure trigger that did not stop status epilepticus but cleared the excess of pilocarpine HCl from the environment (Figure 1A).

**FIGURE 2:**
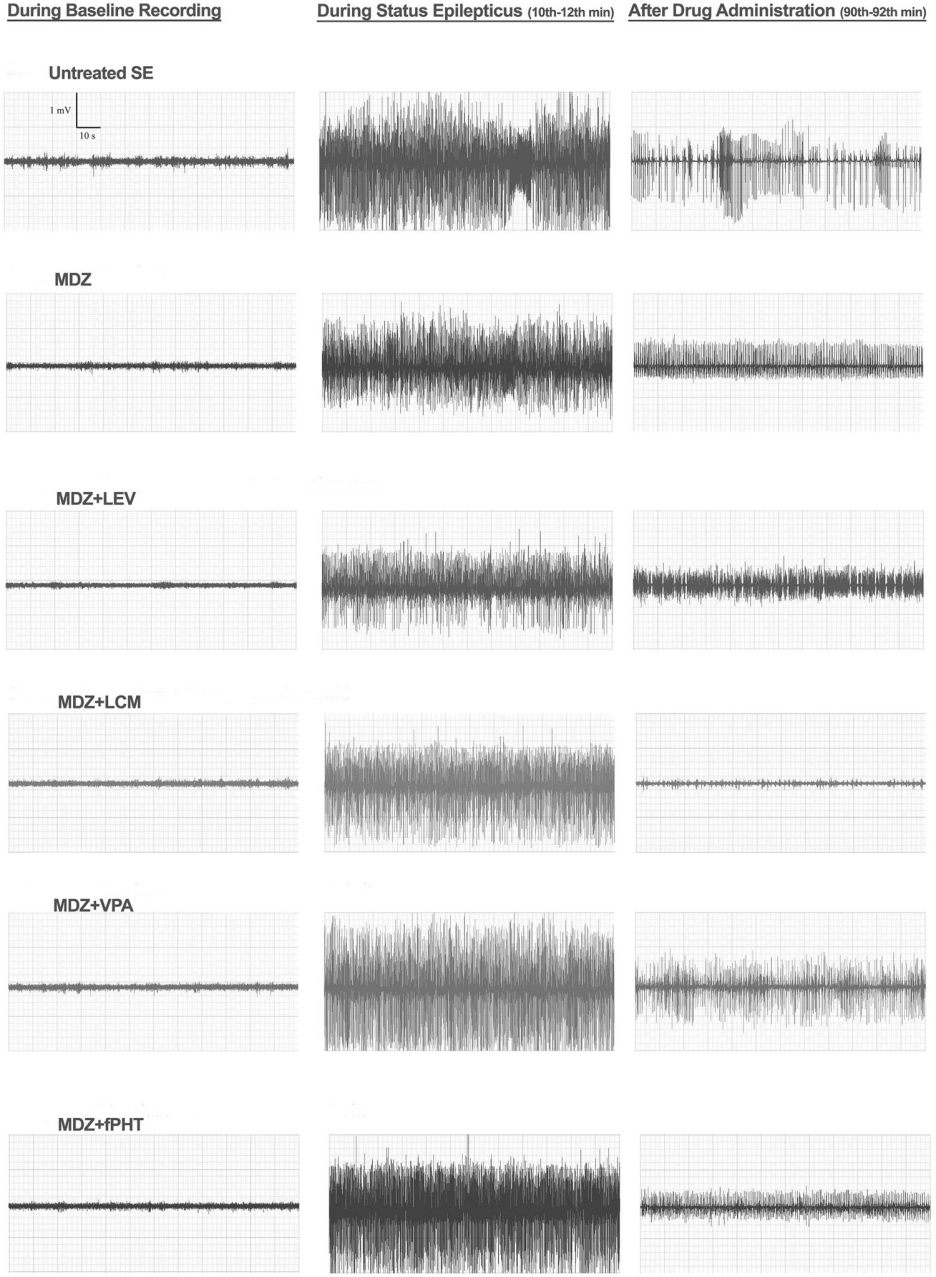
**EEG trace examples of midazolam and antiepileptic drug combinations**. Baseline recordings show 5 min before induction of status epilepticus with pilocarpine. EEG traces during the status epilepticus period show activity between 10 and 12 min after seizures were initiated by pilocarpine injection. The post‐drug period represents activity between 90 and 92 min after administration of MDZ or its combinations. All groups received pilocarpine. Untreated SE: untreated status epilepticus group (*n* = 8); MDZ: midazolam group (*n* = 8); MDZ + LEV: midazolam + levetiracetam group (*n* = 6); MDZ + LCM: midazolam + lacosamide group (*n* = 8); MDZ + VPA: midazolam + valproic acid group (*n* = 6); MDZ + fPHT: midazolam + fosfenitoin group (*n* = 8). EEG, electroencephalography; fPHT, fosphenytoin; LEV, levetiracetam; LCM, lacosamide; MDZ, midazolam; VPA, valproic acid.

The epileptic seizures of animals were evaluated by blind observers according to the behavioral parameters characterized by electrophysiological examination and muscle contractions. The modified Racine scale was used for behavioral scoring of seizures (Nissinen et al. 2000). The Racine scoring scale consists of stages 1–5. According to the modified Racine scale, “score 1” indicates mouth and facial clonus and head shaking; “score 2” indicates clonic jerk of a forelimb; “score 3” indicates bilateral forelimb clonus; “score 4” describes forelimb clonus and rearing; and “score 5” describes forelimb clonus with rearing and falling. Scoring was done during the experiment and checked after the experiment via video‐EEG (Figure 2).

The time taken for the seizure pattern of status epilepticus to begin after pilocarpine injection was defined as the seizure latency. Behavioral seizure parameters (Racine scoring) and electrophysiological recordings were used to determine the seizure latency.

### 2.6 Electrophysiological Recording

Video‐EEG recordings were performed using an electrophysiological data acquisition system (PowerLab 16/35, AD Instruments, Castle Hill, Australia). After the rat was placed in a plexiglass observation cage, the EEG electrode was connected to an amplifier (Animal BioAmp, AD Instruments, Castle Hill, Australia) with a flexible cable. Motor behaviors related to seizures were compared by synchronizing the video image taken with a high‐resolution camera with the EEG. EEG signals were digitized at 1024 Hz, filtered in the range of 0.1–50 Hz, and analyzed using Lab Chart Pro v8.1 software (AD Instruments, Castle Hill, Australia) (Yildirim et al. 2011, 2013; Gedikli et al. 2023).

EEG recordings were made between 09:00 and 16:00. During acute period, video‐EEG monitoring was started to record 30 min of basal activity before status epilepticus and was completed 3 h after drug administration. A total of 4 h of video‐EEG recording was taken. During the analysis phase, LabChart software and analysis modules were used to obtain numerical data regarding epileptic parameters from EEG traces. Seizure onset latency, drug effectiveness latency, spike frequency, and average spike amplitude were converted into numerical data using 4‐h video‐EEG recordings made during the acute experimental study period. During the 2‐week chronic period, 2‐h EEG recordings were performed at 48 h, on Day 7, and on Day 14 from exposure to determine the spike frequency and average spike amplitude of epileptiform activity (Figure 1B).

### 2.7 Behavioral Tests

The open field apparatus is a test apparatus with dimensions of 100 × 100 cm^2^ and a height of 35 cm, made of gray Plexiglas material, in which the locomotor activities of experimental animals can be observed. Experiments were carried out under dim light (40 lux) conditions between 9:00 and 16:00. The animals’ behaviors were monitored for 5 min with a video camera connected to the computer placed on the ceiling of the testing apparatus, and behavioral data were recorded using object tracking software (ANY‐Maze Video Tracking System, Stoelting Europe, Dublin, Ireland). After each test, the apparatus was cleaned with 30% ethyl alcohol. Behavioral parameters of the animals in the open field setup were calculated completely automatically by ANY‐Maze software. The total distance traveled and a number of rearings from the resulting behaviors were evaluated as a measure of locomotor activity. The number of entries to the central area and the time spent in this area were considered indicators of anxiety behavior (Yılmaz et al. 2015; Arslan et al. 2016; Figure 1B).

The Rotarod test apparatus is a mechanism that rotates around itself at certain speeds and is designed so that animals can walk on it (47750 Rota‐Rod NG, Ugo Basile, Italy). Rotarod testing was done in two stages. In the first stage, the rats on the platform were adapted to the Rotarod mechanism by applying a speed of 5 rpm for 1 min. This process was repeated three times with an interval of 10 min. The second phase began 15 min after the first phase was completed. In the second stage, after placing the rats on the Rotarod mechanism moving at a speed of 5 rpm, the speed was increased to 40 rpm, and the time until the rats fell from the rotating cylindrical platform to the floor was determined. For rats that did not fall to the floor and continued to stay on the platform, the procedure was terminated after a maximum of 5 min and the latency was recorded as 5 min. This process was repeated three times with an interval of 15 min. The Rotarod apparatus was cleaned with 30% alcohol after each test (Gedikli et al. 2021, 2023; Figure 1B).

The radial arm maze test apparatus consists of an eight‐arm radial test apparatus used to evaluate spatial learning and memory processes (Yılmaz et al. 2015; İkinci Keleş et al. 2018). The experiment was carried out in three stages: habituation period, learning period, and testing period. During the habituation phase (15th day, not hungry), the rats were allowed to get used to the test apparatus for 10 min. The rat, which fasted for approximately 24 h during the learning phase (16th day, hungry), was placed in the middle section of the test apparatus, where food was placed in only one of the eight arms. The sliding covers of the other arms were closed and then allowed to freely enter and exit the arm containing the food and to be fed for 10 min. After 24 h, the testing phase (17th day, hungry) started. During the testing phase, the rat was released into the device from the middle area, with free access to all arms. The latency to find the arm with the bait in the previous day's learning phase, the number of erroneous arm entries made during this period, and the total distance traveled were determined. Object tracking software (ANY‐Maze Video Tracking System, Stoelting Europe, Dublin, Ireland) was used to record and analyze all behavioral parameters except the animals’ release and retrieval into this test setup. After each test, the entire apparatus was cleaned with 30% ethyl alcohol (Figure 1B).

Passive avoidance mechanism (40552 Passive Avoidance—Step Through, Ugo Basile, Italy) is a behavioral testing device used to examine fear‐based memory. The device can be programmed with the help of software on an accompanying computer (40500‐001 Controller with Touch Screen and 8‐pole Scrambling Shocker Test, Ugo Basile, Italy) and automatically perform all the test stages listed below. The mechanism consists of a bright section and a dark section opening to the bright section with a sliding door. The bright part is made of a transparent material that transmits light, and the floor is illuminated by a light source placed above. In the dark section is a grill made of stainless steel with a diameter of 3 mm, placed at 1 cm intervals, 2 cm above the floor. The animals can be given current from their feet at specified intensities and durations via software from the stimulator, which is connected to this grid and integrated with the computer used as the control unit. The test covers a 2‐day application period. In the learning trial conducted on the first day, the sliding cover separating the two sections was automatically opened 20 s after the rat was placed in the bright section of the apparatus. When the rat completely passed into the dark section, the sliding cover closed automatically, and 0.5 mA electric current was applied to the animal's feet via the stimulator for 3 s. During the test phase, which was carried out 24 h after the learning trial, the rats were placed in the bright area again and were observed for 300 s after the door was opened 20 s later. In this test, the time the rat moved to the dark section was recorded as avoidance latency. All test processes, except placing the animal in the cage and taking it back, were programmed through the software in the control unit, and the data were recorded automatically (40550‐010 Software for Passive Avoidance Test, Ugo Basile, Italy). After each test, the entire apparatus was wiped clean with 30% ethyl alcohol (Yildirim and Marangoz 2004; İkinci Keleş et al. 2018; Figure 1B).

### 2.8 Statistical Analysis

Data obtained from EEG recording, seizure scoring, and behavioral tests and converted into numerical values with the software mentioned in the relevant method sections were evaluated statistically using SPSS v22.0 (IBM Corp., Armonk, NY, USA) software. The suitability of the data for normal distribution was determined by the Shapiro–Wilk test. Because the data did not comply with normal distribution, Mann Whitney *U* test was used after Kruskal Wallis analysis of variance for comparisons between groups. Data are expressed as median (min–max). A *p* value of <0.05 obtained as a result of statistical analysis was considered significant.

## 3 Results

### 3.1 Electrophysiological Findings Related to Status Epilepticus

It was determined that seizure latencies for MDZ and antiepileptic drug combinations of MDZ were not statistically different when compared to the status epilepticus group.

**FIGURE 3:**
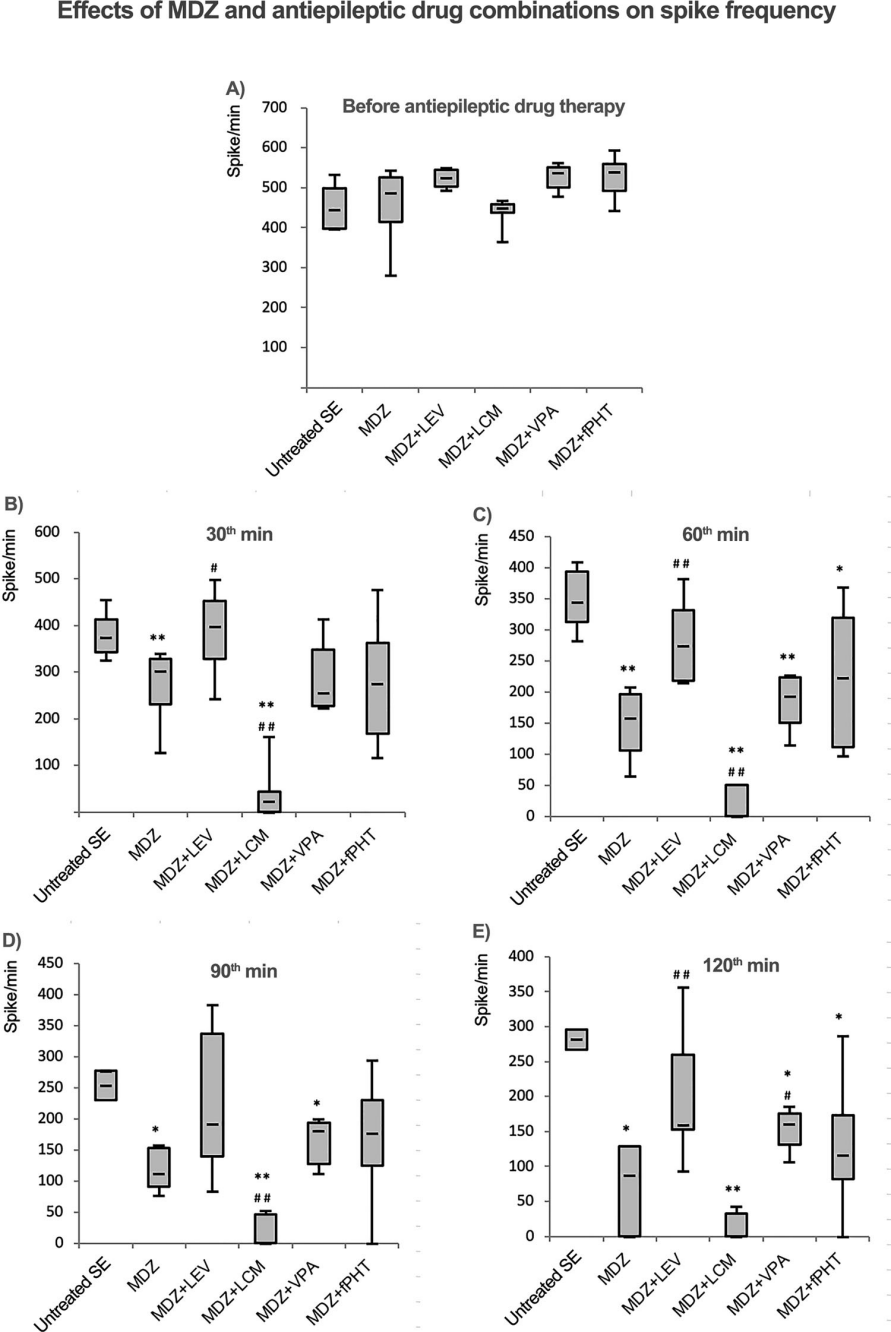
**Spike frequencies related to status epilepticus during the 120‐min EEG recording period before and after the application of MDZ and antiepileptic drug combinations**. Spike frequency values are shown in (A‐E), respectively. Before antiepileptic drug administration (A), at 30th minute (B), 60th minute (C), 90th minute (D) and 120th minute (E). In the experimental groups where status epilepticus was induced with lithium–pilocarpine, 9 mg/kg MDZ alone or in combination with antiepileptic drugs like 200 mg/kg LEV, 50 mg/kg LCM, 300 mg/kg VPA, or 100 mg/kg fPHT were administered intraperitoneally. The graphs show the median (min–max and Q1–Q3) spike frequency values for 1 min of activity, obtained at 30‐min intervals (*p < 0.05 and **p < 0.01 compared to status epilepticus group; #*p* < 0.05 and ##*p* < 0.01 compared to MDZ group. Kruskal Wallis followed by Mann Whitney *U* test). All groups received pilocarpine. Untreated SE: untreated status epilepticus group (*n* = 8); MDZ: midazolam group (*n* = 8); MDZ + LEV: midazolam + levetiracetam group (*n* = 6); MDZ + LCM: midazolam + lacosamide group (*n* = 8); MDZ + VPA: midazolam + valproic acid group (*n* = 6); MDZ + fPHT: midazolam + fosfenitoin group (*n* = 8). EEG, electroencephalography; fPHT, fosphenytoin; LEV, levetiracetam; LCM, lacosamide; MDZ, midazolam; VPA, valproic acid.

EEG trace examples obtained from MDZ and antiepileptic drug combination groups are shown in Figure 2. The median (min–max) spike frequency values at the 30th minute of epileptic activity in the status epilepticus, MDZ, MDZ + LEV, MDZ + LCM, MDZ + VPA, and MDZ + fPHT groups were 445 (397–533), 487 (280–543), 524 (493–551), 449 (364–468), 538 (478–563), and 540 (443–595) spikes/min, respectively. Before the administration of the drugs, it was determined that the spike frequency values in the MDZ and combination groups were not statistically different from the status epilepticus group (Figure 3). Sixty minutes after the application of MDZ or MDZ and antiepileptic drug combinations, median (min–max) spike frequencies in the status epilepticus, MDZ, MDZ + LEV, MDZ + LCM, MDZ + VPA, and MDZ + fPHT groups were 344 (282–409), 157 (64–208), 274 (214–382), 0 (0–51), 193 (115–227), and 223 (97–369) spikes/min, respectively. At the 120th minute, which is the last part of the video‐EEG recording period, the median (min–max) spike frequencies in the status epilepticus, MDZ, MDZ + LEV, MDZ + LCM, MDZ + VPA, and MDZ + fPHT groups were 281 (267–296), 87 (0–129), 159 (93–356), 0 (0–43), 161 (107–186), and 116 (0–287) spikes/min, respectively. It was determined that the spike frequency decreased significantly 30 min after exposure in the MDZ and MDZ + LCM groups and 60 min after exposure in the MDZ + VPA and MDZ + fPHT groups, compared to the status epilepticus group. Especially in the MDZ + LCM group, antiepileptic effects were observed as early as 30 min from exposure. Spike frequency decreased significantly and permanently starting from 30 min after exposure in the MDZ + LCM group compared to the MDZ group (Figure 3).

**FIGURE 4:**
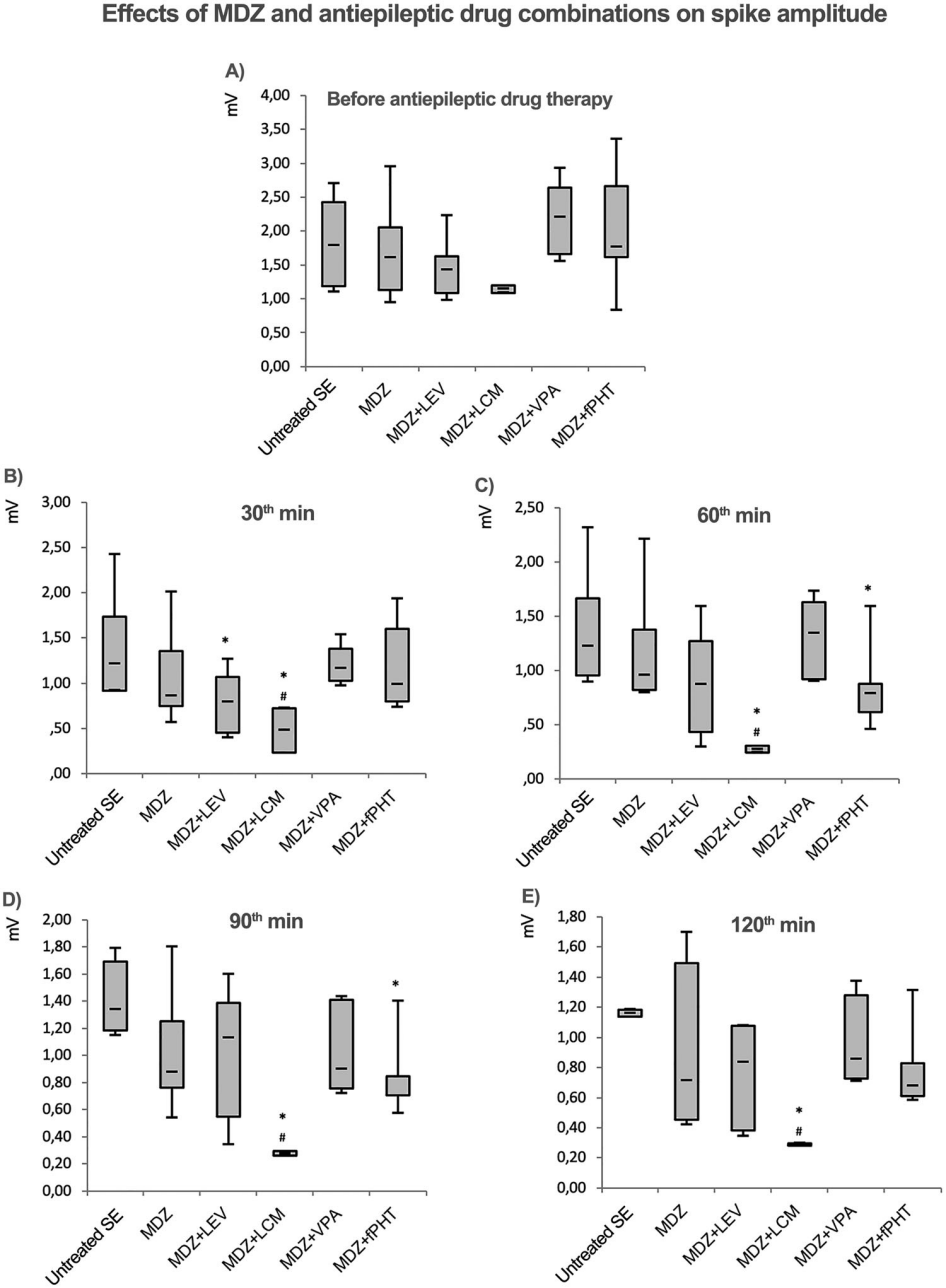
**Spike amplitudes related to status epilepticus during the 120‐min EEG recording period before and after the application of MDZ and antiepileptic drug combinations**. Spike amplitude values are shown in (A‐E) respectively. Before antiepileptic drug administration (A), at 30th minute (B), 60th minute (C), 90th minute (D) and 120th minute (E). In the experimental groups where status epilepticus was induced with lithium–pilocarpine, 9 mg/kg MDZ alone or in combination with antiepileptic drugs like 200 mg/kg LEV, 50 mg/kg LCM, 300 mg/kg VPA, or 100 mg/kg fPHT were administered intraperitoneally. The graphs show the median (min–max and Q1–Q3) spike amplitude values for 1 min of activity, obtained at 30‐min intervals (*p < 0.05 and **p < 0.01 compared to status epilepticus group; #*p* < 0.05 and ##*p* < 0.01 compared to MDZ group. Kruskal Wallis followed by Mann Whitney *U* test). All groups received pilocarpine. Untreated SE: untreated status epilepticus group (*n* = 8); MDZ: midazolam group (*n* = 8); MDZ + LEV: midazolam + levetiracetam group (*n* = 6); MDZ + LCM: midazolam + lacosamide group (*n* = 8); MDZ + VPA: midazolam + valproic acid group (*n* = 6); MDZ + fPHT: midazolam + fosfenitoin group (*n* = 8). EEG, electroencephalography; fPHT, fosphenytoin; LEV, levetiracetam; LCM, lacosamide; MDZ, midazolam; VPA, valproic acid.

Spike amplitude was calculated as the average height of positive (downward) or negative (upward) deflections in the EEG relative to 1 min of epileptiform activity in each 10 min. Median (min–max) spike amplitude values at the 30th minute of epileptic activity in the status epilepticus, MDZ, MDZ + LEV, MDZ + LCM, MDZ + VPA, and MDZ + fPHT groups were 1.79 (1.1–2.7), 1.6 (0.9–2.9), 1.43 (0.9–2.2), 1.14 (1–1.2), 2.2 (1.5–2.9), and 1.77 (0.8–3.3) mV/min, respectively. It was found that the spike amplitude values in MDZ and combination groups did not differ statistically from the status epilepticus group before the administration of drugs (Figure 4). Sixty minutes after the application of MDZ or antiepileptic drug combinations, median (min–max) spike amplitudes in the status epilepticus, MDZ, MDZ + LEV, MDZ + LCM, MDZ + VPA, and MDZ + fPHT groups were 1.22 (0.8–2.3), 0.96 (0.7–2.2), 0.87 (0.3–1.6), 0.27 (0.2–0.3), 1.34 (0.9–1.7), and 0.8 (0.5–1.6) mV/min, respectively. At the 120th minute, which is the final segment of the video‐EEG recording, median (min–max) spike amplitudes in the status epilepticus, MDZ, MDZ + LEV, MDZ + LCM, MDZ + VPA, and MDZ + fPHT groups were 1.16 (1.1–1.2), 0.71 (0.4–1.7), 0.83 (0.4–4.7), 2.9 (0.3–0.3), 0.86 (0.7–1.4), and 0.68 (0.6–1.3) mV/min, respectively. It was observed that spike amplitude significantly decreased 30 min after exposure in the MDZ + LEV and MDZ + LCM groups and 60 min after exposure in the MDZ + fPHT group, compared to the status epilepticus group. Compared to the status epilepticus group, with this decrease being particularly more effective in the MDZ + LCM group. Spike amplitude decreased significantly and permanently starting from 30 min after exposure in the MDZ + LCM group compared to the MDZ group (Figure 4).

Upon examination of the 120‐min video‐EEG recordings taken at the 48th hour of the experimental study, it was observed that at the 60th minute of the 120‐min recording, the median (min–max) spike frequencies in the MDZ, MDZ + LEV, MDZ + LCM, MDZ + VPA, and MDZ + fPHT groups were 36 (2–75), 23 (3–78), 71 (2–184), 33 (7–47), and 23 (0–78) spikes/min, respectively. At the 120th minute, which is the final segment of the video‐EEG recording, the median (min–max) spike frequencies in the MDZ, MDZ + LEV, MDZ + LCM, MDZ + VPA, and MDZ + fPHT groups were 44 (16–62), 24 (0–96), 47 (0–182), 27 (4–57), and 24 (0–45) spikes/min, respectively (Table 1).

**TABLE 1:** Forty eighth hour spike frequency and amplitude values.

	Spike frequency (spike/min)					Spike amplitude (mV)				
	1st min	30th min	60th min	90th min	120th min	1st min	30th min	60th min	90th min	120th min
MDZ	42 (0–89)	27 (0–89)	36 (2–75)	28 (3–148)	44 (16–62)	0.47 (0.4–0.8)	0.48 (0.3–0.7)	0.48 (0.4–0.7)	0.43 (0.3–0.7)	0.44 (0.4–0.7)
MDZ + LEV	22 (4–80)	20 (3–114)	23 (3–78)	23 (0–130)	24 (0–96)	0.37 (0.3–0.6)	0.38 (0.3–0.6)	0.33 (0.3–0.5)	0.39 (0.3–0.5)	0.37 (0.3–0.5)
MDZ + LCM	55 (6–208)	46 (11–162)	71 (2–184)	51 (0–198)	47 (0–182)	0.44 (0.3–0.6)	0.51 (0.3–0.6)	0.43 (0.3–0.6)	0.33 (0.2–0.7)	0.40 (0.3–0.7)
MDZ + VPA	26 (21–72)	31 (15–43)	33 (7–47)	27 (17–55)	27 (4–57)	0.58 (0.4–1)	0.54 (0.3–0.8)	0.50 (0.4–0.8)	0.55 (0.4–0.9)	0.54 (0.4–0.7)
MDZ + fPHT	29 (0–44)	25 (0–49)	23 (0–78)	37 (0–79)	24 (0–45)	0.43 (0.4–0.7)	0.40 (0.4–0.7)	0.40 (0.4–0.9)	0.43 (0.3–0.8)	0.44 (0.3–0.9)

*Note*: Because the entire SE group died, 48th hour EEG recording could not be performed. Values are expressed as median (min–max). Data were analyzed using the Mann–Whitney test following Kruskal Wallis.

Abbreviations: fPHT, fosphenytoin; LCM, lacosamide; LEV, levetiracetam; MDZ, midazolam; VPA, valproic acid.

In the MDZ, MDZ + LEV, MDZ + LCM, MDZ + VPA, and MDZ + fPHT groups, the median (min–max) spike amplitudes at the 60th minute of the 120‐min EEG recording were 0.48 (0.4–0.7), 0.33 (0.3–0.5), 0.43 (0.3–0.6), 0.50 (0.4–0.8), and 0.40 (0.4–0.9) mV/min, respectively. At the 120th minute, which is the final period of the video‐EEG recording, the median (min–max) spike amplitudes in the MDZ, MDZ + LEV, MDZ + LCM, MDZ + VPA, and MDZ + fPHT groups were 0.44 (0.4–0.7), 0.37 (0.3–0.5), 0.40 (0.3–0.7), 0.54 (0.4–0.7), and 0.44 (0.3–0.9) mV/min, respectively (Table 1).

In the chronic period experimental design context, EEG recordings were successfully obtained only at the 48th hour. Some EEG electrodes were not used due to the separation of the animal from the cranium earlier than planned. EEG recordings scheduled for 14 days could not be conducted on these animals. On Days 7 and 14, data could not be presented for these periods, as the number of animals with EEG recordings in the groups was insufficient to conduct a robust statistical analysis (Figure 1B).

### 3.2 Therapeutic Effectiveness Times and Mortality Values of Drugs for Status Epilepticus

**FIGURE 5:**
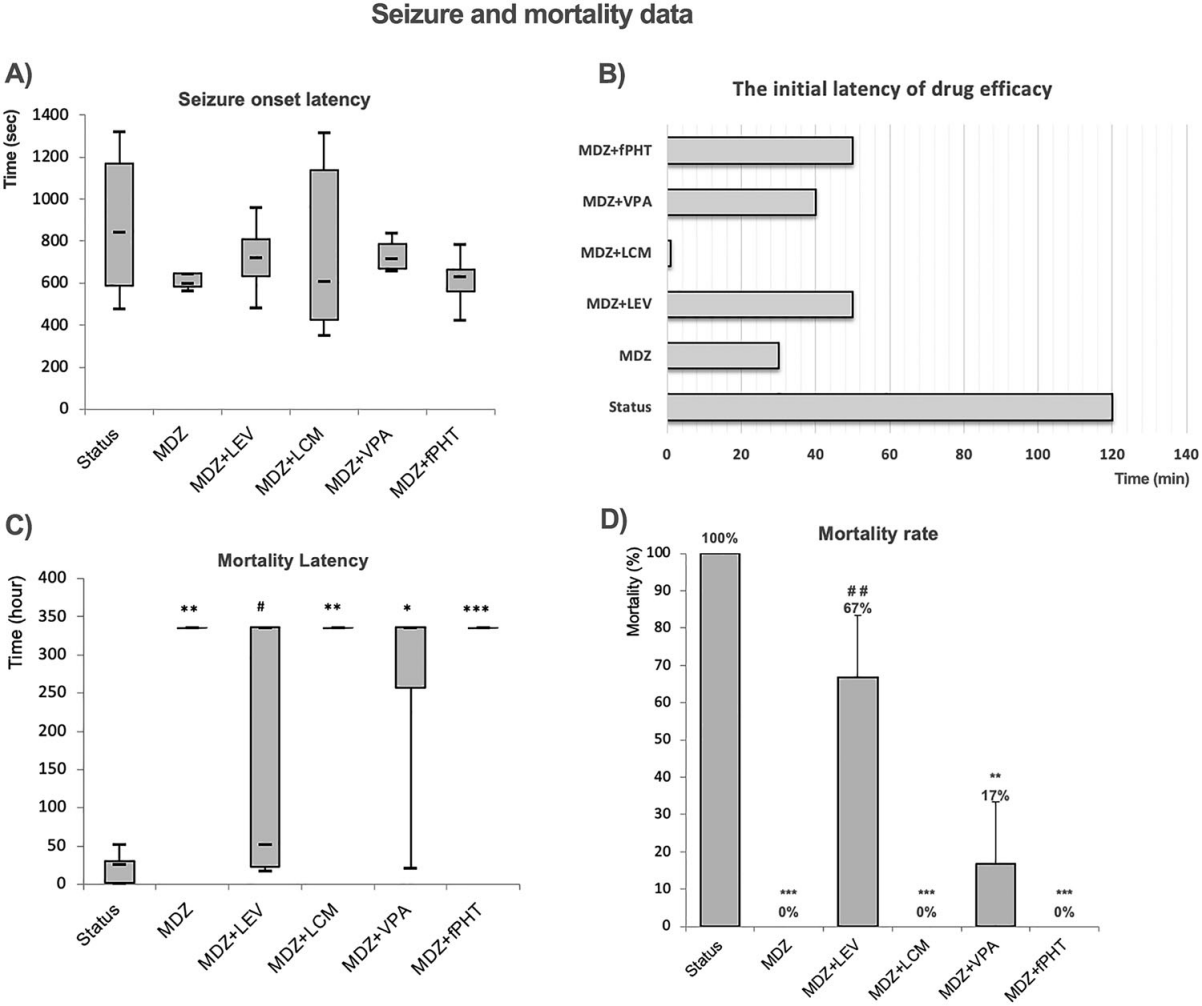
**Effect of MDZ and antiepileptic drug combinations on seizure parameters**. (A) Seizure onset latency shows the median (min–max) values for the time until the onset of seizure activity after 320 mg/kg pilocarpine injection. (B) The initial latency of drug efficacy represents the time until the onset of the first statistically significant decrease in spike frequency, according to the status epilepticus group. (C) Mortality latency shows the median (min–max) values of the animal death time from the physiological saline or drug injections applied at the 30th minute of status epilepticus to the EEG recording on the 14th day. (D) Mortality rate shows the percentage of animal deaths due to status epilepticus in the experimental groups. Because two animals died due to status epilepticus before treatment in the MDZ + LEV and MDZ + VPA groups, the mortality rate in these groups was calculated on *n* = 6 (*p < 0.05, **p < 0.01, and ***p < 0.001 compared to status epilepticus group; #*p* < 0.05 and ##*p* < 0.01 compared to MDZ group. Kruskal Wallis followed by Mann Whitney *U* test). All groups received pilocarpine. Untreated SE: untreated status epilepticus group (*n* = 8); MDZ: midazolam group (*n* = 8); MDZ + LEV: midazolam + levetiracetam group (*n* = 6); MDZ + LCM: midazolam + lacosamide group (*n* = 8); MDZ + VPA: midazolam + valproic acid group (*n* = 6); MDZ + fPHT: midazolam + fosfenitoin group (*n* = 8). EEG, electroencephalography; fPHT, fosphenytoin; LEV, levetiracetam; LCM, lacosamide; MDZ, midazolam; VPA, valproic acid.

When seizure onset latency was examined, it was seen that there was no significant difference between the groups (Figure 5A). Regarding the first efficacy times of drugs, the first time‐point at which the spike frequencies in each experimental group significantly decreased compared to the status epilepticus group was determined as the onset latency of drug efficacy. According to this assessment, the onset latencies of drug efficacy in the MDZ, MDZ + LEV, MDZ + LCM, MDZ + VPA, and MDZ + fPHT groups were 30th, 50th, 10th (immediately after injection), 40th, and 50th minutes, respectively (Figure 5B).

For determining the mortality latencies, the time elapsed after these injections (at the 30th minute of the seizure related to status epilepticus) until the happening of death was used. The mortality latencies were significantly increased in the MDZ, MDZ + LCM, MDZ + VPA, and MDZ + fPHT groups compared to status epilepticus group (*p* < 0.05, 0.01, 0.001), but there was not significance with the MDZ + LEV group. When comparing MDZ with MDZ combinations, MDZ + LEV significantly reduced the mortality latency (*p* < 0.05; Figure 5C).

In determining the mortality rates in the experiment groups, the number of animals dying after the drugs (saline for the status epilepticus group) were injected at the 30th minute of the seizure related to status epilepticus was considered. Compared with the status epilepticus group, the mortality rates were statistically reduced in the MDZ, MDZ + LCM, MDZ + VPA, and MDZ + fPHT groups (*p* < 0.01 or *p* < 0.001). However, it was found that the mortality rate in the MDZ + LEV group did not differ from that in the status epilepticus group. When comparing MDZ with MDZ combination groups, it was observed that the mortality rate significantly increased in the MDZ + LEV group (*p* < 0.01). In contrast, the mortality rates in other combination groups did not differ from the MDZ group (Figure 5D).

### 3.3 Behavioral Tests

The control group did not receive pilocarpine. The SE group was not tested because there was no survival in the SE group, and the MDZ + LEV group was not tested because there were not enough animals left in the MDZ + LEV group.

**FIGURE 6:**
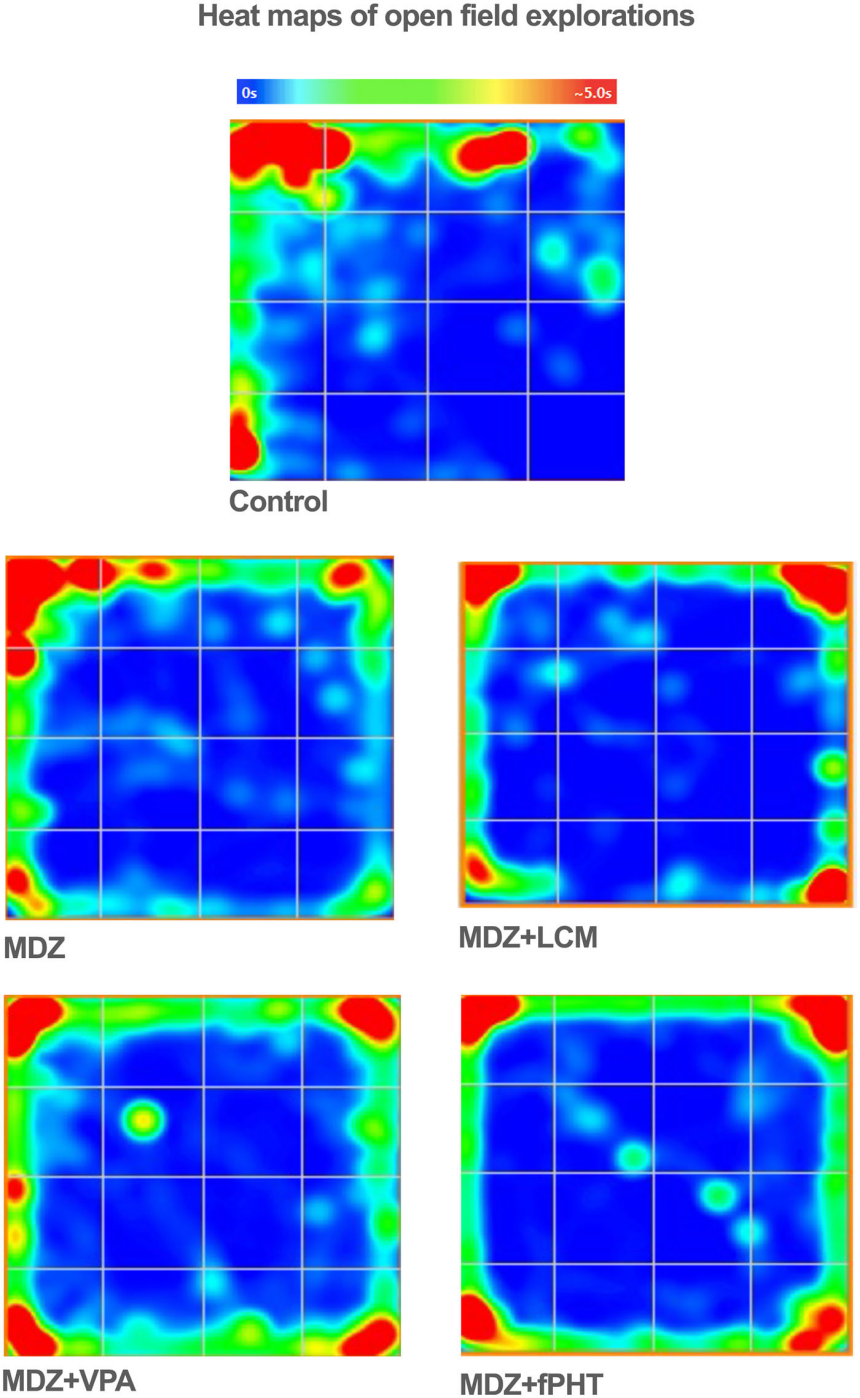
**Heat map depicting the locomotor behavior of animals in an open field test**. A typical representation of the average behavior of each group is shown. The red color highlights areas where more than 5 s were spent. All groups except the control group received pilocarpine. Control: control group (*n* = 8); MDZ: midazolam group (*n* = 8); MDZ + LEV: midazolam + levetiracetam group (*n* = 6); MDZ + LCM: midazolam + lacosamide group (*n* = 8); MDZ + VPA: midazolam + valproic acid group (*n* = 6); MDZ + fPHT: midazolam + fosfenitoin group (*n* = 8). fPHT, fosphenytoin; LEV, levetiracetam; LCM, lacosamide; MDZ, midazolam; VPA, valproic acid.

**FIGURE 7:**
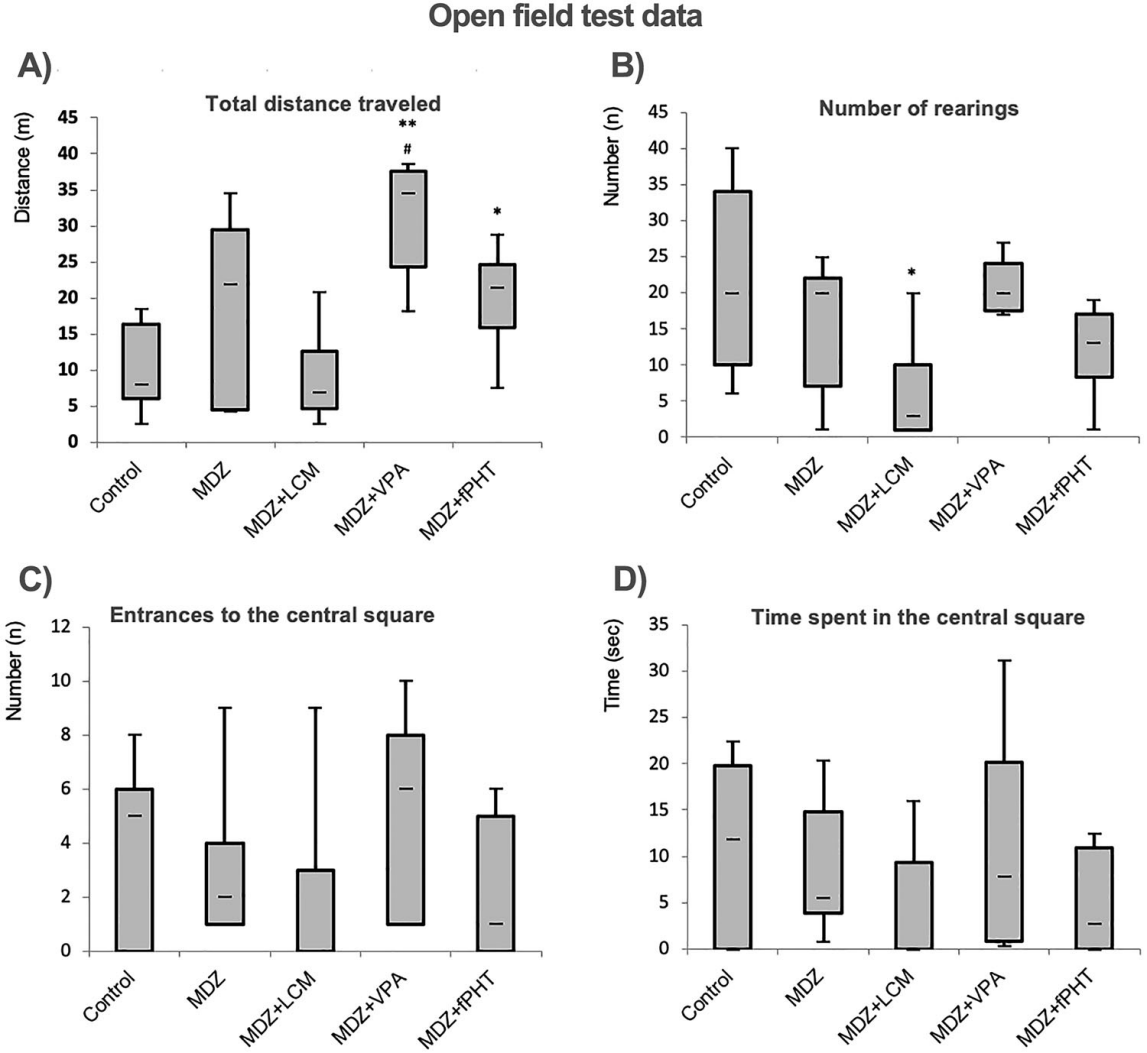
**Behavior parameters for open field testing**. The increase in the total distance traveled (A) and the number of rearings (B) indicates an increase in locomotor efficiency. An increase in the number of times the central square (C) was entered and the time spent (D) there was evaluated as a measure of the decrease in anxiety‐like behavior (*p < 0.05 and **p < 0.01 compared to the control group; #*p* < 0.05 compared to the MDZ group. Kruskal Wallis followed by Mann Whitney *U* test). All groups except the control group received pilocarpine. Control: control group (*n* = 8); MDZ: midazolam group (*n* = 8); MDZ + LEV: midazolam + levetiracetam group (*n* = 6); MDZ + LCM: midazolam + lacosamide group (*n* = 8); MDZ + VPA: midazolam + valproic acid group (*n* = 6); MDZ + fPHT: midazolam + fosfenitoin group (*n* = 8). fPHT, fosphenytoin; LEV, levetiracetam; LCM, lacosamide; MDZ, midazolam; VPA, valproic acid.

For the evaluation of locomotor activity in the open field test, the total distance traveled by the animals, the number of entries into the center squares, the time spent in the center, and the number of rearings were scored using ANY‐Maze software. The locations where the rats spent the most time during the 300‐s test period were converted into heat maps (Figure 6). When compared to the control group, it was found that the total distance traveled by the MDZ + VPA and MDZ + fPHT groups increased (*p* < 0.05 or *p* < 0.01), whereas there was no difference in the distance traveled by the MDZ + LCM group. It was observed that the total distance traveled by the animals in the MDZ + VPA group increased compared to the MDZ group (*p* < 0.05), and there was no difference between the other combination groups and the MDZ group (Figure 7). Only the number of rearings in the MDZ + LCM group decreased compared to the MDZ group (*p* < 0.05), while there was no difference in the number of rearings in the other groups compared to the MDZ group (Figure 7). Additionally, there was no difference in the number of entries into the center squares and the time spent in the center among the combination treatment groups compared to the control and MDZ groups (Figure 7).

**FIGURE 8:**
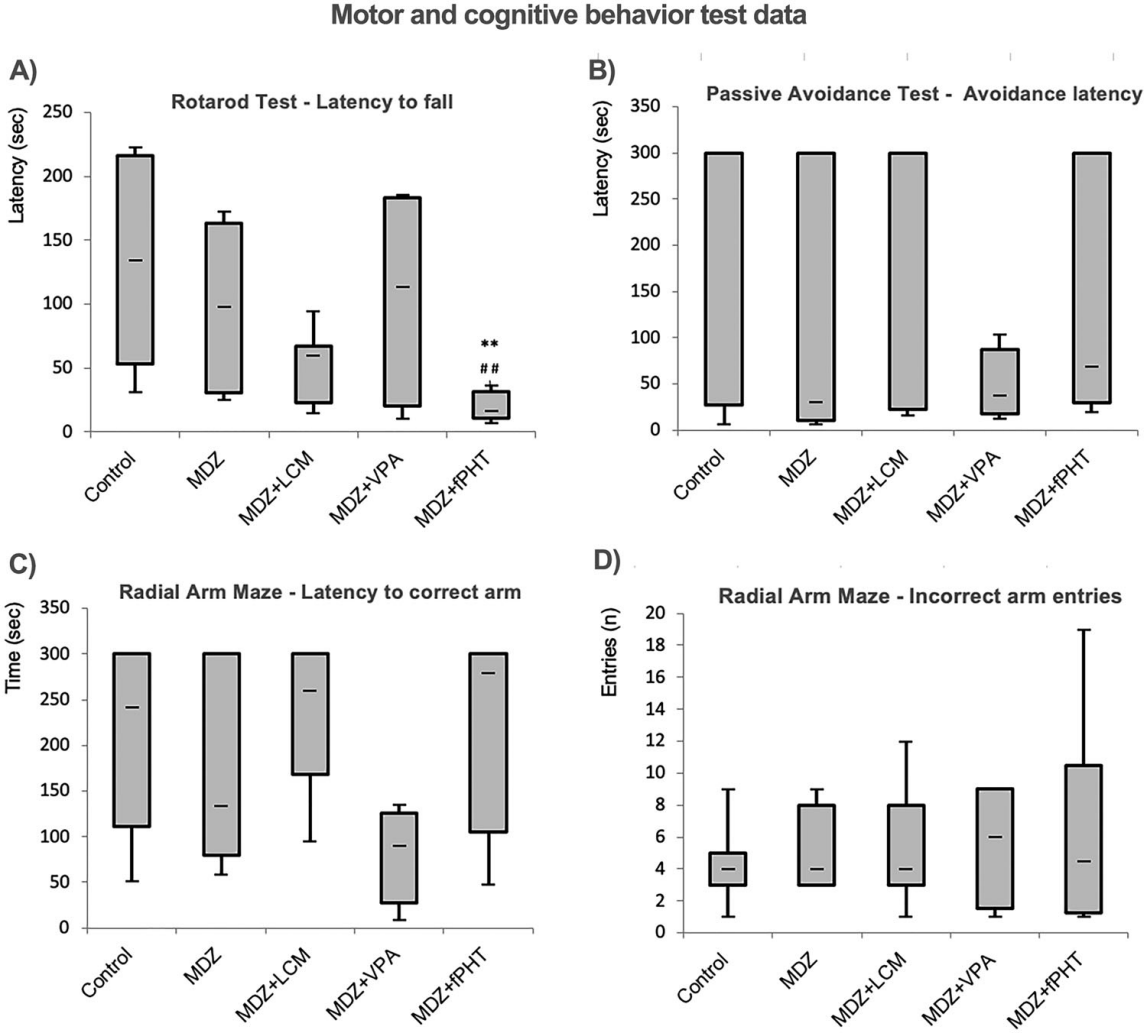
**Motor and cognitive test parameters**. Fall latency in the Rotarod test was assessed as an indicator of forced motor activity (A). Avoidance latency in the passive avoidance test reflects a reduction in fear‐based memory (B). In the radial arm maze test, the latency of locating the correct arm (C) was deemed a favorable parameter for spatial memory, whereas the number of incorrect arm entries (D) was seen as a negative parameter (*p < 0.05 and **p < 0.01 compared to control group; #*p* < 0.05 and ##*p* < 0.01 compared to MDZ group. Kruskal Wallis followed by Mann Whitney *U* test). All groups except the control group received pilocarpine. Control: control group (*n* = 8); MDZ: midazolam group (*n* = 8); MDZ + LEV: midazolam + levetiracetam group (*n* = 6); MDZ + LCM: midazolam + lacosamide group (*n* = 8); MDZ + VPA: midazolam + valproic acid group (*n* = 6); MDZ + fPHT: midazolam + fosfenitoin group (*n* = 8). fPHT, fosphenytoin; LEV, levetiracetam; LCM, lacosamide; MDZ, midazolam; VPA, valproic acid.

In the Rotarod apparatus, which tests motor coordination and grip strength, it was determined that there was a decrease in the time spent on the rotating platform in the MDZ + fPHT group compared to the control and MDZ groups (*p* > 0.01), but there was no significant difference in the other combination treatment groups (Figure 8A).

In the passive avoidance apparatus, compared to the control and MDZ groups, there was no difference in the passive avoidance latencies of the antiepileptic drug combination treatment groups (Figure 8B).

In the radial arm maze test, when compared with the control and MDZ groups, there was no statistically significant difference in the latency of finding the correct arm and the number of entering the incorrect arm in the antiepileptic drug combination treatment groups (Figure 8C,D).

## 4 Discussion

In the presented study, it was observed that when administered simultaneously in combination with MDZ during the acute phase after the onset of status epilepticus, LCM, VPA, and fPHT resulted in a statistically significant decrease in mortality and spike frequency compared to the untreated status epilepticus group. However, when LEV was administered with MDZ, the favorable effects achieved with the other three polytherapies could not be attained. When compared to MDZ monotherapy, it was found that MDZ + LEV increased mortality and spike frequency (*p* < 0.01), whereas MDZ + LCM spike frequency (*p* < 0.01) and amplitude (*p* < 0.05) decreased. MDZ combined with VPA only spike frequency decreased (*p* < 0.01), with no difference observed in other parameters. No statistically significant difference was found between MDZ monotherapy and MDZ + fPHT polytherapy in any of the seizure or mortality parameters.

Experimental and clinical studies examining new approaches to the treatment of status epilepticus are available in the literature. In a clinical study, the efficacy of PHT, VPA, and LEV in second‐line therapy was investigated, reporting no statistical difference between VPA and PHT. At the same time, LEV was found to be less effective than the other two groups (Alvarez et al. 2011). In a study conducted on 60 patients, the majority with convulsive status epilepticus, VPA provided seizure control in 66% and PHT in 42% of cases. When used as a second option, VPA provided control in 79% of cases and PHT in 25%. It was concluded that VPA provided stronger seizure control than PHT (Misra et al. 2006). Another experimental study published by Efendioglu et al. (2020) showed that 50 mg/kg LEV reduced the intensity, frequency, and total duration of seizures facilitated by PTZ in adult male Sprague–Dawley rats with mild traumatic brain injury, but this positive effect on seizure‐related parameters was abolished when LEV was administered with *N*‐acetylcysteine. In a study by Niquet. et al. (2016) on rats, there was no significant difference in the management and mortality rates of lithium–pilocarpine‐induced status epilepticus between groups treated with MDZ alone and MDZ + VPA. In the same status epilepticus model induced by lithium–pilocarpine, DZP + VPA + ketamine triple polytherapy has been shown to be much more effective in suppressing status epilepticus compared to DZP, VPA, or ketamine monotherapy administered at doses 3–5 times higher (Niquet et al. 2017). Additionally, it has been found that simultaneous administration of lower doses of 3 mg/kg MDZ, 30 mg/kg ketamine, and 90 mg/kg VPA as polytherapy instead of sequential administration at 30‐min intervals is more effective in the treatment of status epilepticus. In the same studies, double and triple polytherapy of MDZ, VPA, and ketamine were tested in the status epilepticus model created with lithium–pilocarpine, and it was reported that simultaneous double polytherapy was more effective than sequential monotherapy and triple polytherapy was more effective than double polytherapy (Niquet et al. 2019a, 2019b; Lumley et al. 2021).

In the presented study, when the effects of MDZ monotherapy and MDZ in combination with antiepileptic drugs (LEV, LCM, VPA, and fPHT) were evaluated together on electrophysiological and behavioral parameters, it was found that MDZ + LCM polytherapy was more effective in controlling status epilepticus than MDZ monotherapy. It was also determined that MDZ + fPHT polytherapy was not different from MDZ monotherapy; however, MDZ + VPA and MDZ + LEV polytherapy were not as effective as MDZ application alone. MDZ + VPA polytherapy was found to be effective compared to the untreated SE group. Consistent with the presented findings, studies support the use of LCM as a component of polytherapy in status epilepticus. For example, in a clinical study, it has been found that if LCM is used earlier during treatment (as the second or third antiepileptic drug), it is more effective in terminating status epilepticus compared to those not given LCM (Santamarina et al. 2013). It has been also reported that LCM is more effective when given as the third/fourth drug (72%) than when given as the fifth or subsequent drug (56%) (Miro et al. 2013). In an experimental study in rats, LCM administered in combination with ketamine and MDZ was reported to be more effective in combating soman‐induced seizures, epileptogenesis, and brain pathology than MDZ monotherapy or dual therapy with MDZ and LCM (Lumley et al. 2024). When the studies in the literature are evaluated as a whole, it has been stated that LCM is more effective in controlling status epilepticus than other antiepileptic drugs in both monotherapy and polytherapy. The findings obtained from the presented study regarding the effectiveness of MDZ + LCM polytherapy were found to be compatible with the literature. However, the presented study is not a dose–response study testing different doses of used antiepileptic drugs but only reflects the efficacy of a single dose of each antiepileptic drug selected following the literature. In future studies, examining the effectiveness of lower doses in the combined use of antiepileptic drugs will be useful in terms of literature.

MDZ has been shown to act as a positive allosteric modulator that increases GABA_A_ receptor‐mediated chloride conductance in neuronal cell‐cultured and brain tissue slices (Alsbo et al. 2001). It has been stated that LCM can control pathophysiological neuronal hyperexcitability by selectively increasing the slow inactivation of voltage‐gated sodium channels (Carona et al. 2021). It has also been suggested that, similar to other sodium channel blockers, it may also be effective in the fast inactivation process of voltage‐gated sodium channels but has slower binding kinetics (Jo and Bean 2017). Interestingly, LCM has been shown to target GABA_A_ receptors and act synergistically with LEV to reduce use‐dependent GABA dysfunction, which is considered one of the hallmarks of drug‐resistant epilepsies, in cortical brain tissues from patients with refractory epilepsy (Ruffolo et al. 2018). According to the presented study, MDZ + LCM combination therapy was found to be the most effective polytherapy option in reducing seizures and mortality in the lithium–pilocarpine‐induced status epilepticus model. It appears that the combination of MDZ + LCM provides effective seizure control in lithium–pilocarpine‐induced status epilepticus by a possible mechanism of enhancing GABAA receptor‐mediated synaptic inhibition and inactivation of voltage gated sodium channels.

In the presented study, it was determined that antiepileptic drugs (LEV, LCM, VPA, and fPHT) administered in combination with MDZ did not cause a negative effect on learning and memory compared to both the control group and the MDZ group, but fPHT administration together with MDZ impaired the motor functions (*p* < 0.01). In addition to providing seizure control, it has been stated that LCM has secondary positive effects on cognitive function and behavior in children and adolescents and that this positive effect does not remain at a secondary level but provides a primary increase in cognitive function such as increasing the speed of information processing (Moavero et al. 2017). In the presented study, no behavioral disorders were detected in the combination of LCM with MDZ.

## 5 Conclusions

According to the findings of the presented study, MDZ + LCM combination therapy was found to be the most effective polytherapy option in reducing seizures and mortality in the lithium–pilocarpine‐induced status epilepticus model. MDZ + LEV polytherapy was found to be less effective than MDZ monotherapy in reducing seizures and mortality. In conclusion, it was determined that the most effective polytherapy option among current experimental applications was MDZ + LCM, and MDZ monotherapy, MDZ + VPA, and MDZ + fPHT treatment options were also effective in reducing seizures and mortality.

## Author Contributions

**Cumaali Demirtas**: conceptualization, investigation, writing – original draft, data curation, methodology, funding acquisition, visualization. **Metehan Akca**: investigation, writing – original draft. **Ugur Aykin**: investigation, writing – original draft, visualization. **Yunus Emre Surmeneli**: investigation. **Hava Yildirim**: investigation, writing – original draft. **Mehmet Yildirim**: conceptualization, investigation, writing – review and editing, supervision, methodology, project administration, funding acquisition, visualization.

## Ethics Statement

All application and experimental study procedures regarding animals were approved by the SBÜ Hamidiye Animal Experiments Local Ethics Committee (Decision no.: 2020‐03/15).

### Peer Review

The peer review history for this article is available at https://publons.com/publon/10.1002/brb3.70546

## Data Availability

The data that support the findings of this study are available from the corresponding author upon reasonable request.
